# Prolonged Amphetamine Exposures Increase the Endogenous Human Dopamine Receptors 2 at the Cellular Membrane in Cells Lacking the Dopamine Transporter

**DOI:** 10.3389/fncel.2021.681539

**Published:** 2021-08-26

**Authors:** Vindhya Nawaratne, Sean P. McLaughlin, Felix P. Mayer, Zayna Gichi, Alyssa Mastriano, Lucia Carvelli

**Affiliations:** ^1^Department of Biology, Harriet L. Wilkes Honors College, Florida Atlantic University, Jupiter, FL, United States; ^2^Brain Institute, Florida Atlantic University, Jupiter, FL, United States

**Keywords:** amphetamine, dopamine receptors, G-protein coupled receptors, cAMP, dopamine transporter

## Abstract

The dopamine 2 receptors (D2R) are G-protein coupled receptors expressed both in pre- and post-synaptic terminals that play an important role in mediating the physiological and behavioral effects of amphetamine (Amph). Previous studies have indicated that the effects of Amph at the D2R mainly rely on the ability of Amph to robustly increase extracellular dopamine through the dopamine transporter (DAT). This implies that the effects of Amph on D2R require the neurotransmitter dopamine. However, because of its lipophilic nature, Amph can cross the cellular membrane and thus potentially affect D2R expression independently of dopamine and DAT, *e.g.*, in post-synaptic terminals. Here we used an *in vitro* system to study whether Amph affects total expression, cellular distribution, and function of the human D2R (hD2R), endogenously expressed in HEK293 cells. By performing Western blot experiments, we found that prolonged treatments with 1 or 50 μM Amph cause a significant decrease of the endogenous hD2R in cells transfected with human DAT (hDAT). On the other hand, in cells lacking expression of DAT, quantification of the hD2R-mediated changes in cAMP, biotinylation assays, Western blots and imaging experiments demonstrated an increase of hD2R at the cellular membrane after 15-h treatments with Amph. Moreover, imaging data suggested that barbadin, a specific inhibitor of the βarrestin-βadaptin interaction, blocked the Amph-induced increase of hD2R. Taken together our data suggest that prolonged exposures to Amph decrease or increase the endogenous hD2R at the cellular membrane in HEK293 cells expressing or lacking hDAT, respectively. Considering that this drug is often consumed for prolonged periods, during which tolerance develops, our data suggest that even in absence of DAT or dopamine, Amph can still alter D2R distribution and function.

## Introduction

Amphetamine (Amph) is a psychostimulant broadly prescribed as long-term therapy for attention deficit hyperactivity disorder (ADHD). Moreover, Amph and Amph-like drugs are commonly abused as recreational drugs and performance enhancers. Despite decades of active research, the exact molecular mechanism(s) of Amph and its analogs remain far from being completely understood. Numerous studies have shown that Amph alters the function of various proteins in the brain, and, among others, proteins linked to the dopamine signaling are major targets. In fact, *in vivo* and *in vitro* experiments have shown that proteins expressed in dopaminergic neurons, such as the dopamine transporter (DAT) and the type 2 dopamine receptors (D2R) are altered during and after Amph treatments (Sulzer, 2011; Ashok et al., 2017). Because dopamine is highly involved in the action of addictive drugs – all drugs of abuse increase synaptic dopamine – it is not surprising that this drug alters the function of DAT and D2R. In fact, these two proteins are key players of dopamine transmission. DAT, for example, by restricting the spatial and temporal action of dopamine and, thus, ensuring fast clearance of extracellular dopamine, is a direct target of Amph. As matter of fact, Amph is a DAT substrate through which Amph gets quick access inside the neurons. The intracellular accumulation of Amph causes several detrimental consequences *e.g.*, reduction of the transporters at the cellular membrane (Saunders et al., 2000), depletion of vesicular dopamine stores and reverse transport of dopamine through DAT (Carvelli et al., 2010; Sulzer, 2011), ultimately causing an increase of DAT- and vesicle-mediated (Daberkow et al., 2013) dopamine release.

Since Amph increases extracellular dopamine, one anticipated consequence of continuous dopamine overflow in the synaptic cleft is the indirect effect of Amph at the dopaminergic receptors. The D2R are located at both pre- and post-synaptic neuronal terminals and the D2R-containing pre-synaptic terminals express DAT as well. Previous findings revealed that the D2R are involved in the reinforcing properties of drugs of abuse (Andrianarivelo et al., 2019), and animals lacking expression of the D2R exhibit reduced Amph-induced behaviors (Carvelli et al., 2010; Solís et al., 2021). Recent studies in human beings also reported that Amph users exhibit significant changes in D2/D3 receptors availability (Ashok et al., 2017). Taken together, these data strongly suggest that the D2R play an important role in mediating the physiological and behavioral changes caused by Amph.

As with most G-protein coupled receptors (GPCR), the activity of the D2R can be regulated by desensitization. Continuous stimulation leads to uncoupling of the receptor from the G-protein, binding to arrestin and receptor internalization (Gainetdinov et al., 2004). While the Amph-induced redistribution of DAT from and to the cell membrane has been well documented (Saunders et al., 2000; Boudanova et al., 2008; Lute et al., 2008; Daberkow et al., 2013; Wheeler et al., 2015), the effects of Amph on D2R redistribution are controversial. For example, some groups have shown an increase in striatal D2R after a 14-day regimen (Levy et al., 1988) or repeated daily treatments (Kilbourna and Dominob, 2011) with Amph, whereas others have found a decrease (Kamata and Rebec, 1984) or no change (Bonhomme et al., 1995). Moreover, Calipari et al. (2014) showed that 5 days of *ad libitum* Amph intake reduced the ability of quinpirole to inhibit dopamine release and, simultaneously, abolished the interaction between the pre-synaptic D2R and the GPCR subunit Gαi2, in rat midbrain, but not in the striatum. These results suggest that Amph affects the D2R located in some, but not all, areas of the brain expressing the D2R (Calipari et al., 2014). The diverging results reported above might reflect the complexity of Amph action, particularly, when this drug is investigated in complex animals such as murine models. For example, the effects of Amph at the D2R located in post-synaptic terminals could be different than those observed in pre-synaptic terminals. To overcome these limitations, we performed a set of experiments to test *in vitro* whether D2R function and distribution were altered following prolonged treatments with physiologically relevant concentrations of Amph. Using a cell line which endogenously expresses human D2R (hD2R), we found that when cells were transfected with hDAT, 15 h of continuous exposure to Amph significantly decreased the expression of endogenous D2R. On the other hand, cells devoid of hDAT exhibited an increase of endogenous D2R in the cellular membrane in absence of dopamine. In these cells, the Amph-induced increase of D2R was confirmed using surface biotinylation assays and confocal microscopy imaging. Also, functional data showed that after D2R activation, the forskolin-mediated cAMP production was significantly decreased in samples treated with Amph for 15 h, again suggesting an increased number of functional D2R at the cellular membrane in cells lacking expression of hDAT and in absence of dopamine. Thus, our results show that Amph, because of its lipophilic nature, might directly affect D2R distribution during prolonged treatments.

## Materials and Methods

### Cell Cultures

Human embryonic kidney 293T (HEK293) cells were cultured in Dulbecco’s Modified Eagle’s Medium (DMEM; 10569-010, Gibco) supplemented with 10% fetal bovine serum (FBS 16000-4, Gibco) and 1% penicillin/streptomycin (15140-122, Gibco). To confirm the specificity of the D2R antibody or for our cAMP assays, HEK293 cells were transiently transfected with 2.5 μg cDNA of Flag-D2R using TransIT^®^-LT1 Transfection Reagent (MIR2305, Mirus). The Flag-D2R cDNA was a generous gift from Dr. Jonathan Javitch – Columbia University. Twenty-four hours after transfection, cells were treated with control or Amph for 15 h. HEK293 cells stably expressing hDAT (gift from Randy Blakely – Florida Atlantic University) were additionally supplemented with 250 ug/ml G418 (30-234-Cl, Corning) to maintain the selection pressure. Cells were dissociated using 0.25% trypsin diluted with versene (2 mM EDTA in PBS) and maintained in a humidified incubator with 5% CO_2_ at 37°C.

### Western Blot Assays

HEK293 cells (0.9 – 1.1 million cells/well) were seeded in 6-well plates. After 24 h, cells were treated with control, 1 or 50 μM Amph for 15 h, then collected using versene (2 mM EDTA in PBS) and counted. Six million cells from each treatment group were solubilized with 100 μL of lysis buffer (20 mM Hepes pH 7.4, 100 mM NaCl, 1.5 mM MgCl2, 1 mM EDTA, 1 mM EGTA, 0.1% NP40, and proteases/phosphatases inhibitors), passed through a 29-gage needle 25 times and centrifuged for 10 min at 12,000 × *g*. Total amount of proteins in the supernatant was quantified with Pierce^TM^ BCA Protein Assay Kit (23225, Thermo Scientific^TM^). Lysates (30 μg) were denatured with sample buffer containing 20 mM DTT for 1 h, loaded and run on SDS-PAGE gels at 120 V for 2 h and the proteins transferred into PVDF membranes (IPVH00010, Millipore) at 35 V for 16 h at 4°C. The blots were first exposed to the hD2R antibody (1:1000, AB5084P, Sigma-Aldrich) and then detected with an anti-rabbit HRP (1:3000, 65-6120, Invitrogen) and ECL (34577, Thermo Scientific^TM^). The same blots were then probed with a β-actin antibody (1:2000, sc-69879, Santa Cruz) and detected with an anti-mouse IRDye^®^ 680RD (1:10000, 925-68072, LI-COR Biosciences). Images were quantified using Image Studio LI-COR Biosciences. For each gel, sample data were normalized to β-actin bands. Because the amount of D2R with higher molecular weights (75–130 kDa) was more concentrated than those at lower molecular weights (40 kDa), PVDF membranes had different exposure times during band acquisition. This guaranteed a detectable signal for the bands at lower molecular weights, and at the same time, the upper bands were not overexposed.

### Confocal Microscopy

Confocal microscopy was performed using a Nikon A1R confocal microscope in the FAU-Brain Institute using a 60× oil-immersion objective and Nikon capture software. HEK293 cells were seeded at 40,000 cells/well in 8 well glass bottom μ-slides (80827, Ibidis) and grown over night. After treating with control, 1 μM or 50 μM Amph with or without 20 μM barbadin for 15 h, cells were fixed for 20 min at room temperature with 3.5% formaldehyde (28906, Thermo Scientific^TM^) prepared in 50% phosphate-buffered saline (PBS), 4% sucrose and 2.5 mM CaCl_2_. Samples were kept at room temperature for the rest of the experiment. After fixation, cells were washed 3 times with PBS, blocked with 1% BSA in PBS, and stained for 90 min with Anti-Na^+^/K^+^ ATPase α-1 antibody (05-369, Sigma-Aldrich) diluted to 1:200. Cells were then washed with PBS 3 times to eliminate any unbound antibody and permeabilized with 0.1% saponin and 0.2% BSA in PBS for 30 min. Saponin solution was removed, and cells were incubated for 90 min with the D2R antibody (AB5084P, Sigma-Aldrich) at a final dilution of 1:1000. Samples were washed 3 times with PBS, incubated with anti-mouse Alexa Fluor 405 (A31553, Invitrogen) and anti-rabbit Alexa Fluor 546 (A10040, Invitrogen) secondary antibodies for 30 min. Alexa Fluor 405 and anti-rabbit Alexa Fluor 546 were stimulated using 405 and 561 laser lines, respectively, and detected through DAPI and TRITC filters, respectively. Acquisition of the two fluorophores was done sequentially to prevent cross talk. 2D optical sections of 0.2 or 0.5 μm were acquired using a Nikon A1R HD Galvano scanner. Image deconvolution, figure imaging and fluorescence quantification were performed with FIJI software (ImageJ, NIH). For quantification analysis of the D2R at the cellular membrane and cytoplasm, images of the Na^+^/K^+^ ATPase antibody (yellow stain) were used to define the cell surface as regions of interest (RoI). These RoI were overlayed onto the images of the same cells labeled with the D2R antibody (cyan stain). Max fluorescence values of the cellular membrane (the RoI) and the cytoplasm (the intracellular region) were measured with ImageJ. Max fluorescence values were used instead of mean fluorescence because some of our cell images (Figures 2D–K) exhibited one or more black areas (most likely the nucleus) within the cytoplasm. Inclusion of these black areas in our quantification would have created random artifacts in our analyses. Fluorescence values are reported as the average of at least 3 independent experiments ± SEM.

**FIGURE 1:**
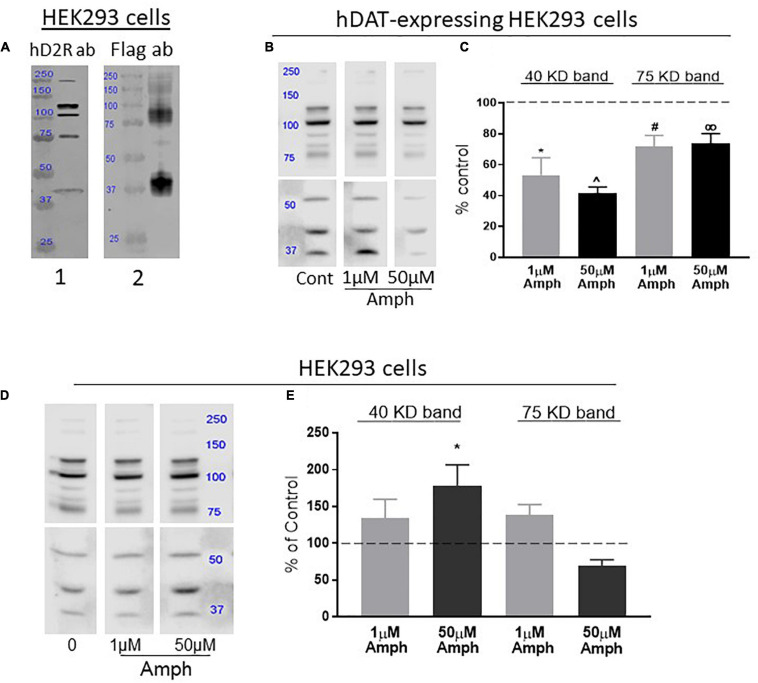
Amphetamine (Amph) decreases the overall expression of the endogenous D2R in cells transfected with hDAT but increases D2R in cells lacking hDAT. **(A)** Antibodies against human D2R identify distinct bands in lysates from HEK293 cells (panel 1). Lysates from HEK293 cells transfected with Flag-D2R and probed with Flag antibodies show two prominent bands at 40 and 75 KD (panel 2). These bands match in size with bands probed with the hD2R antibody in panel 1. **(B)** A representative gel from hDAT-transfected HEK293 cells treated with control, 1 or 50 μM amphetamine (Amph) and immunoblotted with hD2R antibodies is shown, and the average of 4 independent Western blot experiments is plotted in **(C)**. Statistical analysis against control-treated samples was performed using one-way ANOVA Dunnett’s multiple comparisons test (*p = 0.0007, ^*p* = 0.0001, ^#^*p* = 0.01, ^∞^*p* = 0.03, each compared to controls). **(D)** A representative gel of samples from HEK293 cells treated with control, 1 or 50 μM Amph and immunoblotted with hD2R antibodies is shown, and the average of 4 independent Western blot experiments is plotted in **(E)**. PVDF membranes from Western blots in **(B,D)** are shown as two halves because different times of exposure to ECL were used. This guaranteed detectable and quantifiable signals at both lower and higher molecular weight bands (see section “Materials and Methods”). Statistical analysis was performed using one-way ANOVA Dunnett’s multiple comparisons test (*p = 0.04).

**FIGURE 2:**
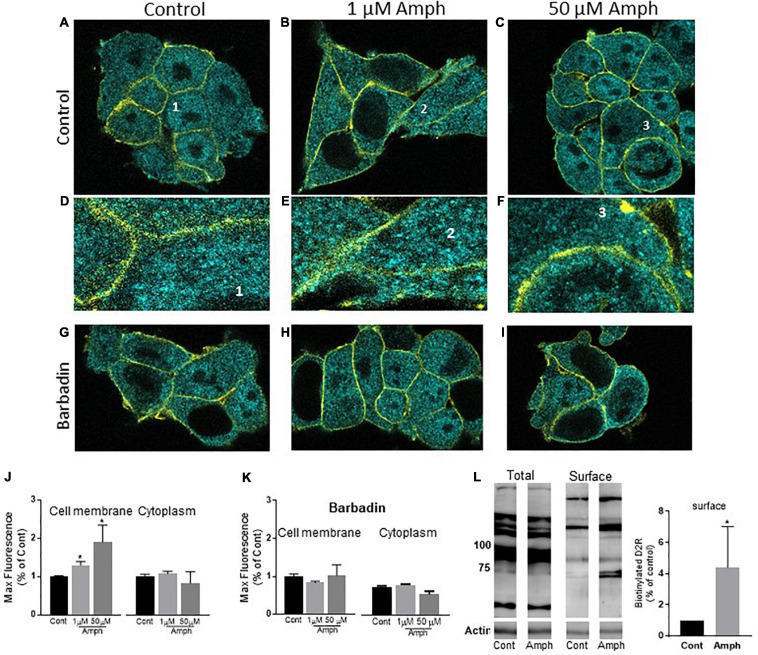
Prolonged amphetamine treatments increase the endogenous hD2R at the cellular membrane in HEK293 cells. **(A–C)** representative confocal images of control **(A)**, 1 **(B)**, or 50 **(C)** μM Amph treated cells shown as 0.2 μm sections for the cyan channel (D2R stain) and the yellow channel (Na^+^/K^+^-ATPase stain). The latter highlights the cellular membrane of each cell. Details of a single cell from **(A–C)** (1–3) are magnified in **(D–F)**. Representative cells treated with 20 μM barbadin alone or barbadin together with 1 or 50 μM Amph are shown in **(G–I)**, respectively. **(J)** Average of max fluorescence of cyan puncta from 3 independent experiments shows Amph increases D2R at the cellular membrane but not in the cytoplasm (*p = 0.03, one-way ANOVA test). **(K)** Averages from 3 independent experiments show 20 μM barbadin prevents Amph-induced increase of max fluorescence at the cellular membrane. **(L)** Biotinylated samples (Surface) show increased D2R band intensity after 15-h treatment with 1 μM Amph with respect to control treated cells (*p = 0.04, *t*-test). One representative gel is shown of 4 independent experiments.

### Cell Surface Biotinylation Assays

After 15-h treatments with/without 1 μM Amph, proteins at the cellular membrane of approximately 8 million HEK293 cells, grown in PDL coated T-75 flasks, were biotinylated with the membrane-impermeant reagent Sulfo-NHS-LC-Biotin (Thermo Fisher, Cat. No 21335) in PBS containing 1 mM MgCl_2_, 0.1 mM CaCl_2_ (PBS^2+^) for 45 min at 4°C. Cells were subsequently washed twice with ice cold PBS^2+^, lysed in RIPA buffer (supplemented with 1% Triton X-100 and 1% protease inhibitors - Millipore Sigma, Cat No. P8340), and cleared by centrifugation (15 min at 13,000 × *g*). To separate biotinylated proteins from non-biotinylated proteins, 600-1800 ug of total protein were exposed to NeutrAvidin agarose beads (ThermoFisher, Cat No. 29201) for 16 h at 4°C at constant nutation. Prior to the addition of protein, beads were washed 3 times with ice-cold RIPA buffer (supplemented with 1% Triton-X 100 and protease inhibitors). The next day, the supernatant (flow through) was removed from the beads and stored on ice and the beads were washed 3 times with ice-cold RIPA buffer (supplemented with 1% Triton-X 100 and protease inhibitors). Subsequently, proteins were eluted by subjecting the beads to 240 μL of Laemmli sample buffer for 45 min at room temperature on a laboratory agitator (TOMY, MT-360). 220 μL of sample buffer containing proteins obtained from the pulldown, flow through and 15 μg of the corresponding total protein, were separated on SDS-PAGE gels (10% polyacrylamide) for 2 h at 120 V. Finally, protein bands were probed using the same primary/secondary antibodies and conditions as described for our Western blot protocol, except the D2R antibody was used at a 1:200 dilution to compensate for the reduced number of proteins collected in the pulldown.

### cAMP Assays

cAMP-Glo^TM^ Assay kit (V1502, Promega) was used to detect intracellular cAMP. HEK293 cells were seeded (900,000 cells/well) in 6-well plates, grown overnight, and transfected with 2.5 μg cDNA of the human Flag-D2R receptor (gift from Johnathan Javitch) using TransIT^®^-LT1 Transfection Reagent (MIR2305, Mirus). After 24 h, cells were treated with control, 1 μM or 50 μM Amph in absence or presence of 10 nM haloperidol for 15 h. Cells were collected using versene (2 mM EDTA in PBS), resuspended in serum-free media containing 100 μM isobutyl-1-methylxanthine (I5879, Sigma-Aldrich) and 100 μM 4-(3-butoxy-4-methoxybenzyl) imidazolidone (B8279, Sigma-Aldrich), and plated in a 96-well plate (200,000 cells/well). To stimulate adenylate cyclase, we incubated the cells with 3–100 μM forskolin (F6886, Sigma-Aldrich) in absence or presence of 100 μM of the D2R agonist sumanirole (SML1087, Sigma-Aldrich) for 15 min at 37°C. Cells were lysed following the assay kit’s instructions and luminescence was detected following the manufacturer’s protocol (cAMP-Glo^TM^ Assay kit). Relative light units were converted to cAMP using a cAMP standard curve and normalized to basal cAMP. Concentration-response curves for forskolin in the absence and presence of sumanirole were fitted with the three-parameter non-linear regression model. Two-way ANOVA with Bonferroni’s multiple comparisons test was performed to determine statistical significance.

### Dopamine ELISA Assay

An enzyme-linked immunosorbent assay (ELISA, LDN, Nordhorn, Germany) was performed to quantify levels of intracellular dopamine in HEK293 cells. About 4 million cells were grown in DMEM media at 37°C. Cells were detached with 0.25% of trypsin, lysed using radioimmunoprecipitation assay (RIPA) buffer, collected in microcentrifuge tubes and then sonicated for 30 s prior to centrifugation at 13,000 RPM for 1 min. The samples and the provided standards from the kit were pipetted into a 48-well extraction plate and acylated as per the manufacturer’s instructions. The resulting supernatant was collected and pipetted into a 96-well microtiter plate for enzymatic conversion, incubated for 2 h and then transferred to pre-coated dopamine microtiter strips. After treating the samples with rabbit dopamine antiserum overnight at 4°C, the antiserum was discarded, then replaced with goat anti-rabbit immunoglobulins. Lastly, a chromogenic substrate was added. Absorbance was measured using a FLUOstar^®^ Omega microplate reader (BMG LABTECH, Orenberg, Germany). A standard curve was generated using a four-parameter logistic regression, and absorbance readings were plotted on the resulting curve to determine dopamine concentrations. Dopamine concentrations reported in the Results section are average of 2 independent experiments ± SEM, and each experiment was done in quadruple.

### Statistical Analysis

GraphPad Prism 7.05 was used for statistical analysis and only *p*-values smaller than 0.05 were considered statistically significant. Specific statistical tests are detailed in each graph (see section “Results” or “Figure Legends”).

## Results

### Continuous Exposure to Amphetamine Reduces Endogenous Expression of the Human D2 Receptors in Cells Expressing the Dopamine Transporter

Previous publications have reported conflicting data about the ability of Amph to induce reallocation of the D2R to and from the cellular membrane (Kamata and Rebec, 1984; Levy et al., 1988; Bonhomme et al., 1995; Kilbourna and Dominob, 2011). As most of these experiments were performed using *in vivo* or *ex vivo* preparations, we reasoned simpler preparations, such as cell cultures, might help us clarify these diverging results. We chose to use human embryonic kidney cells (HEK293) because they express endogenous hD2R (Bowton et al., 2010); and, since specific antibodies against these receptors are commercially available, we designed a set of experiments with the goal to quantify changes in endogenous hD2R expression following Amph exposure.

We validated the efficacy of the D2R antibody in our assays by performing parallel experiments where samples from HEK293 cells probed with the hD2R antibody were compared to samples from HEK293 cells transfected with Flag-hD2R and probed with an antibody against the Flag. Similar to previous data obtained in HEK293 cells transfected with D2R (Free et al., 2007), cell lysates from parental HEK293 cells probed with the hD2R antibody showed multiple bands (Figure 1A, panel 1). These bands most likely account for D2R monomers, dimers and multimers, and perhaps, also different levels of protein glycosylation (Free et al., 2007). On the other hand, the Flag-hD2R expressing cells probed with the Flag antibody showed two prominent bands, at around 40 and 75 kDa, and a faded smear of bands between 100 and 250 kDa (Figure 1A, panel 2). Because the bands at 40 and 75 KD were also present in samples probed with the hD2R antibody (Figure 1, compare panels 1,2), we focused our analysis on these two bands which, most likely, represent D2R monomers (40 kDa) and possible dimers (75 kDa) (Free et al., 2007).

D2R and DAT are co-expressed in the pre-synaptic terminals of dopaminergic neurons and transport through DAT is the main route of Amph for fast access into the cytoplasm of these neurons. Thus, we investigated the effects of 15-h Amph exposures on D2R in HEK293 cells, which were previously engineered to stably express hDAT. Western blot experiments demonstrated that cell lysates from hDAT-expressing HEK293 cells exhibit multiple D2R bands arranged in a pattern similar to that observed in HEK293 cells (Figure 1B) and, also in these cells, the 40 and 75 kDa bands were detected. Importantly, we found that samples from hDAT-expressing cells treated with 1 or 50 μM Amph for 15 h, exhibited a significant decrease in the 40 kDa band (53.3 ± 11.3 and 41.6 ± 3.9%, respectively) with respect to the control-treated cells (Figures 1B,C; one-way ANOVA Dunnett’s multiple comparisons test). Similarly, both Amph concentrations reduced the 75 kDa band to 77 ± 7 and 71 ± 5%. These results demonstrate a significant reduction in endogenous hD2R expression following 15-h exposure to 1 or 50 μM Amph in HEK293 cells expressing hDAT.

### Continuous Exposure to Amphetamine Increases Endogenous Expression of the Human D2 Receptors in HEK293 Cells Lacking hDAT

The D2R are expressed in pre-synaptic terminals alongside DAT and in post-synaptic terminals which do not express DAT. We investigated if Amph could affect expression of the hD2R in cells lacking DAT expression. Although less effective than via transporter-mediated access, Amph can cross the cellular membrane via diffusion due to its lipophilic nature (Zaczek et al., 1991). Thus, we investigated whether 15-h Amph treatments could affect the endogenous expression of the hD2R receptors in HEK293 cells which do not express hDAT. After treating the cells with control solution, 1 or 50 μM Amph, cell lysates were separated by SDS-PAGE, transfered onto PVDF membranes and probed with hD2R antibodies. Surprisingly, we found that while 1 μM Amph generated a moderate, but not statistically significant, increase of 38 ± 23 and 39 ± 14% at the 40 and 75 kDa bands, respectively, the increase caused by 50 μM Amph at the 40 kDa band was more robust and statistically significant (78 ± 29%; ^∗^*p* < 0.05, one-way ANOVA Dunnett’s multiple comparisons test; Figures 1D,E). No significant change was seen at the 75 kDa band after 15-h treatment with 50 μM Amph. Taken together these results show that 15-h exposure to Amph increases expression of the endogenous hD2R monomer in cells that do not express hDAT.

### Amphetamine Enhances Endogenous D2 Receptors at the Cellular Membrane of Cells Lacking hDAT and in Absence of Dopamine

Our Western blot results (Figure 1) show that the effects of Amph at the D2R are dictated by DAT, *i.e.*, cells expressing hDAT exhibit reduction in D2R following prolonged Amph treatments whereas, cells lacking hDAT exhibit an increase of D2R. We were particularly intrigued by the observation that Amph altered the D2R in absence of DAT. Thus, we continued our studies using HEK293 cells which endogenously express the hD2R but not DAT.

While the data shown in Figures 1D,E indicate that Amph increases expression of endogenous D2R, it remains elusive whether these changes occurred in specific sub-compartments of the cell, *e.g.*, cellular membrane vs. cytoplasm or both. Using confocal microscopy, we investigated the Amph-induced changes in hD2R expression at the cellular membrane and in the cytoplasm by collecting images from cells stained for hD2R and Na^+^/K^+^-ATPase. The latter was used as a marker of the cellular membrane. As for our Western blot assays, cells were treated with control solution, 1 or 50 μM Amph for 15 h before being fixed and probed with the two antibodies. Cells exhibited a well-defined Na^+^/K^+^-ATPase staining which was confined to the cellular membrane (Figures 2A–F, yellow stain) and, in accordance with previous publications (Prou et al., 2001), the hD2R antibody revealed puncta all over the cell with most of the puncta located in cytoplasmic compartments (Figures 2A–F, cyan stain). The yellow stain of the Na^+^/K^+^-ATPase was used to identify the area of the cell membrane for quantification analysis of the D2R. Cyan puncta included in this area were selected as D2R in or at closed proximity of the cellular membrane. The cyan puncta outside this area were selected as D2R located in the cytoplasm. Figures 2A–C are representative confocal images of 0.2 μm sections from cells treated for 15 h with control (A,D), 1 μM (B,E), or 50 μM (C,F) Amph. Figures 2D–F display selected areas (labeled 1–3) from Figures 2 A–C respectively, at higher magnification. Magnified images and quantification of fluorescence signals from three independent experiments, showed an increase in D2R at the cellular membrane after 15-h exposure to 1 or 50 μM Amph with respect to controls (Figure 2J, left panel. Compare E,F to D). No significant change was measured in the cytoplasm between control- and Amph-treated cells (Figure 2J, right panel). It should be noted that our imaging data showed that the 15-h treatment with Amph did not alter the Na^+^/K^+^-ATPase expression (yellow stain) with respect to control-treated cells (2798 ± 423 and 2353 ± 296 max fluorescence, respectively) suggesting, therefore, that Amph does not alter the expression of every protein at the cellular membrane. Moreover, as previously shown (Ferdous et al., 2020), we did not observe any obvious cytotoxic effect induced by the 15 h treatment with both Amph concentrations. In fact, cell morphology (Figures 2A–C) and the number of cells in control-treated samples (14 ± 3 cells/microslide) were comparable to those treated with 1 or 50 μM Amph (13 ± 2 or 13 ± 3 cells/microslide, respectively). Taken together these results show that, in cells lacking expression of hDAT, 15-h exposure to 1 or 50 μM Amph does not affect cell proliferation but causes an increase of endogenous hD2R at the cellular membrane.

For most G-protein coupled receptors, including the D2R, the magnitude of the receptor-mediated signal can be modulated by moving receptors to and from the cellular membrane, a mechanism known as trafficking. Trafficking of clathrin-coated vesicles forming at the cellular membrane (endocytosis) or in intracellular organelles (exocytosis) is one of the major mechanisms used by cells to shuffle proteins at the cellular membrane (Jamieson and Palade, 1971; Pearse, 1976). Among others, β-arrestin and adaptor proteins (AP) are essential proteins for facilitating vesicle budding. Recently, Beautrait et al. (2017) identified a specific drug, barbadin, which prevents the agonist-induced trafficking of G-protein coupled receptors by selectively blocking the interaction between β-arrestin and the AP2 subunit β-adaptin. Thus, we reasoned to test whether the Amph-induced changes at the endogenous D2R could be affected by barbadin. Barbadin treatments did not alter cell morphology (Figure 2, compare G–I with A–C) or cell proliferation. In fact, 15 h treatment with barbadin alone or together with 1 or 50 μM Amph yielded the same number of cells (13 ± 2, 15 ± 1, or 12 ± 2 cells/microslide, respectively) with respect to control-treated samples (14 ± 3 cells/microslide). Moreover, barbadin did not change the quantity of D2R with respect to controls at the cellular membrane (682 ± 96 vs. 701 ± 45, respectively), nor in the cytoplasm (782 ± 55 vs. 784 ± 87, respectively). Interestingly though, barbadin completely blocked the Amph-induced increase of D2R at the cellular membrane (left panel in Figures 2K,G–I). No change in fluorescence intensity was measured in the cytoplasm after barbadin and Amph treatment (right panel in Figures 2K,G–I). Taken together these data demonstrate that Amph enhances D2R at the cellular membrane and inhibition of the β-arrestin/β-adaptin interaction prevents this effect.

Our confocal experiments were replicated consistently over multiple independent experiments. Yet, imaging data are snapshots of a relatively small number of cells and artifacts, such as cell-membrane overlay cannot be excluded. Hence, we supported our studies with a biochemical approach. Biotinylation of cell surface proteins is a technique allowing to discriminate proteins at the cellular membrane from those in the cytoplasm by tagging the surface-expressed proteins with the membrane impermeable reagent Sulfo-NHS-LC-Biotin. We used this technique to further confirm the data collected from our imaging experiments. As the expression of D2R at the cellular membrane is very low with respect to the cytoplasm (Prou et al., 2001), about 8 million cells, pretreated with control or 1 μM Amph, were exposed to Sulfo-NHS-LC-Biotin. This high number of cells, which yielded approximately 1,800 μg of proteins, ensured the detection of D2R at the cell surface. Using avidin beads, we separated biotinylated proteins (proteins at the cell surface) from the proteins in the cytosol. Samples from the biotinylated protein fraction were separated by SDS-PAGE and then immunoblotted with the D2R antibody. As shown in Figure 2L, 1 μM Amph treatment for 15 h increased the band intensity of the D2R at the cell surface (^∗^*p* = 0.04; *t*-test. *N* = 4). These results, once again, demonstrate that prolonged exposure to low concentrations of Amph enhances the quantity of endogenous D2R at the cellular membrane in HEK293 cells. It is worth noting that our pulldown (membrane surface) samples did not show D2R bands lower than 75 KD (Figure 2L, representative gel). This might suggest that only mature (glycosylated) and multimeric D2R reside at the cellular membrane.

Our data show that prolonged treatments with 1 or 50 μM Amph affect hD2R distribution in cells lacking hDAT, thus, suggesting that Amph permeates the cellular membrane of these cells or enters the cells via a DAT-independent mechanism. In this scenario, one could imagine that Amph, after depolarizing these cells, would cause dopamine release into the media and, in turn, dopamine would affect hD2R distribution on the cellular membrane. For this to be true, one should prove that HEK293 cells indeed produce dopamine. We used an Elisa kit (DNL), which is designed for high-sensitive quantification of dopamine, to quantify dopamine in HEK293 cells. Lysed samples from cells, which were grown and treated following the same conditions as our Western blot and imaging experiments, exhibited a concentration of dopamine that was equivalent or below our blank samples (0.003 ± 0.06 and 0.005 ± 0.02 ng/mL, respectively). Instead, and as expected, our positive control, pure dopamine included in the Elisa kit, showed a concentration of dopamine equal to 5.5 ± 1.5 ng/mL (*N* = 6). Moreover, our HEK293 cells were grown and treated in media/serum which did not contain dopamine. Taken together, these data demonstrate that the effects of Amph on the D2R are not mediated by dopamine.

### Continuous Exposure to Amphetamine Reduces Intracellular cAMP Through the Activation of the D2 Receptors

**FIGURE 3:**
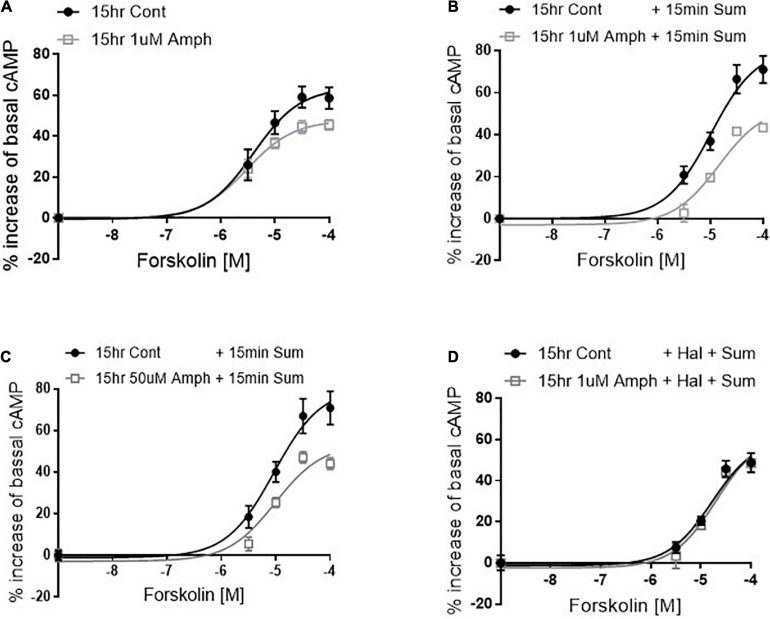
Amphetamine reduces forskolin- and D2R-induced cAMP production. After pretreating cells with control or 1 μM amphetamine (Amph), cells were treated with increasing concentrations of forskolin **(A)**. cAMP production was statistically lower in Amph-pretreated samples with respect to controls (*p* = 0.006). Parallel samples were treated for 15 min with 100 μM sumanirole (Sum) after 15-h pretreatment with control, 1 μM **(B)** or 50 μM **(C)** Amph. Amph significantly reduced cAMP production (*p* = 0.0001). Coincubation of 10 nM haloperidol (Hal) with 1 μM Amph prevented the Sum-induced decrease of cAMP **(D)**. Each graph is the average of 3 independent experiments. Statistical analysis was performed with 2-way ANOVA Bonferroni multiple comparison test.

So far, our data demonstrate that in cells expressing endogenous hD2R but lacking dopamine and hDAT, prolonged exposure to Amph increases hD2R at the cellular membrane (Figures 1, 2). Next, we examined whether the enhancement of the D2R caused by Amph might be of physiological relevance. Because the D2R couple to inhibitory G-protein subunits, which reduce cAMP synthesis by blocking adenylate cyclase, we tested whether the Amph-induced changes at the D2R altered intracellular cAMP production. We could not detect the inhibition of forskolin-mediated cAMP production when we activated the endogenous hD2R, most likely because of the low number of receptors at the membrane. Thus, we transfected our cells with hD2R. As expected, in hD2R transfected cells, stimulation with different concentrations of the adenylate cyclase activator forskolin caused an increase of intracellular cAMP (Figure 3A, black circles). Interestingly, 15-h exposure to 1 μM Amph caused a statistically significant decrease of cAMP with respect to cells pretreated with control solution (Figure 2A gray squares; *p* = 0.005, two-way ANOVA test). These results suggest that prolonged Amph exposures reduces the intracellular production of cAMP. However, they did not sufficiently support the conclusion that this effect is mediated by the D2R. Therefore, we tested whether activation of the D2R in cells treated with Amph would produce different levels of cAMP with respect to control-treated cells. After cells were pretreated for 15 h with control, 1 or 50 μM Amph, we thoroughly washed out the drug and treated the cells for 15 min with the D2R agonist sumanirole. We stimulated the D2R with sumanirole because, in contrast to dopamine, this compound is a highly specific D2R agonist (McCall et al., 2005). As shown in Figures 3B,C, cells pretreated with 1 or 50 μM Amph exhibited statistically lower levels of intracellular cAMP with respect to cells pretreated with control solution during forskolin activation (*p* = 0.0001, two-way ANOVA Bonferroni multiple comparison test), whereas sumanirole alone did not cause a statistically different response to forskolin with respect to control treated cells (compare filled circles of Figure 3B with A). Importantly, the Amph-induced effect was prevented when haloperidol, a specific D2R antagonist, was co-applied (Figure 3D). These results demonstrate that prolonged Amph treatments increase the D2R-induced inhibition of cAMP and, therefore, they suggest that Amph upregulates the D2R and/or increases the number of functional D2R at the cellular membrane.

## Discussion

Amphetamine is a psychostimulant broadly used as a performance enhancer and is one of the main drugs prescribed to treat attention deficit disorders. Therapeutical doses of Amph, which are in the range of low μM, improve concentration in people suffering with attention disorders. Amph is also one of the most popular “study drugs” used by college students. Very often though, students end up taking higher doses to extend the benefit of the drug during exam taking or because tolerance develops overtime. The physiological consequences of these higher doses taken for longer and consecutive periods are unknown. Here, we developed an *in vitro* model to study the effects of 15-h exposure to 1 and 50 μM Amph in HEK293 cells. We chose these cells because they endogenously express one of the major targets of Amph, the type 2 dopaminergic receptors (D2R). These receptors play an important role in mediating the physiological and behavioral effects of Amph (Centonze et al., 2001; Seneca et al., 2006; Fan and Hess, 2007; Nelson and Killcross, 2013; Calipari et al., 2014) and, previous reports demonstrated that, in animal models as well as in human studies, D2R expression and function are altered by chronic exposure to Amph (Chen et al., 1999; Calipari et al., 2014; Ashok et al., 2017).

The D2R are expressed in the pre-synaptic terminals, where they colocalize with another major target of Amph, DAT, and in post-synaptic terminals. While it has been suggested that D2R and DAT reciprocally regulate each other (Bolan et al., 2007; Lee et al., 2007; Bertolino et al., 2009), and thus, might cooperatively mediate the effects of Amph, it is not clear if Amph directly alters the D2R in cells lacking expression of DAT. Although less efficiently than via carrier-mediated transport, Amph can cross cellular membranes because of its lipophilicity (Zaczek et al., 1991). Thus, in situations where relatively high concentrations and prolonged exposures to Amph occur, it is reasonable to assume that Amph can affect D2R function in cells lacking expression of DAT in presence or absence of dopamine. For this reason, we investigated whether prolonged Amph exposure altered the endogenous expression of the D2R in HEK293 cells. We recognize that cell cultures do not fully reproduce the physiological conditions seen in *in vivo* models. Nonetheless, they allow us to study the action of a drug in a well-defined pathway without the complexity and interference of other signaling. Moreover, because specific ligands or antibodies capable of discriminating between pre- and post-synaptic D2R do not exist, our model allowed us to reproduce an *in vitro* scenario in which Amph acts at the D2R without the influence of DAT and/or dopamine.

Previous data have suggested that the Amph-induced internalization of D2R was a consequence of the increased extracellular dopamine caused by Amph (Sun et al., 2003). For example, Skinbjerg et al. (2010) showed that Amph causes robust reductions of radioligands binding at the D2R which persisted for several hours (4–24) after the Amph injection. However, extracellular dopamine returned to basal levels within 2 h. Hence, it was concluded that the dopamine surge seen after Amph application would induce internalization of the D2R (Skinbjerg et al., 2010). Yet, this study could not discriminate whether the D2R internalization caused by Amph occurred in pre- or post-synaptic cells. Here, we designed an *in vitro* model showing that the effects of Amph on the endogenous D2R are different in parental cells vs. cells transfected with hDAT. Our Western blot data showed that in hDAT-expressing cells, prolonged Amph treatments cause a strong reduction of the endogenous D2R in absence of dopamine. On the other hand, in cells lacking both DAT and dopamine, Amph generated an increase of D2R at the cellular membrane (Figure 2). This result was in agreement with previous *in vivo* data showing increased binding potential of the D2R antagonist [^11^C]raclopride in rats after repeated Amph treatments (Kilbourna and Dominob, 2011). The effect of Amph at the D2R in cells lacking both DAT and dopamine is novel and interesting because it suggests that prolonged use of low concentrations of Amph might directly affect D2R distribution in post-synaptic terminals or in cells expressing D2R but not DAT. Interestingly, we found that blocking the interaction between β-arrestin and β-adaptin, a subunit of the AP2 complex, completely prevented the Amph-induced increase of D2R at the cellular membrane. These data suggest that the recruitment of D2R to the membrane is mediated by clathrin-coated vesicles.

In accordance with data obtained using HEK293 cells transfected with hD2R-Flag (Free et al., 2007), our results show that the hD2R endogenously expressed in these same cells exhibit multiple bands when separated in SDS gels (Figure 1) suggesting, therefore, that glycosylation and/or dimerization are integral processes of hD2R expression (Free et al., 2007). Moreover, Free et al. (2007) performed several experiments, and convincingly proved that the bands at 40 and 75 kDa represent un-glycosylated D2R. Interestingly, our results show that these same bands are predominantly affected by the 15-h treatment with Amph. Together these data suggest that lack of glycosylation at the D2R might be required by Amph to affect the expression of the D2R at the cell membrane. Future studies including drugs known to specifically inhibit protein glycosylation will determine whether Amph-induced changes at the D2R are inhibited by blocking D2R glycosylation.

Data collected in this study also suggest that the Amph-induced increase of D2R at the cellular membrane can generate physiological effects since prolonged treatments with Amph reduced the intracellular concentration of cAMP. D2R couple to G proteins of the Gi/o subfamily which inhibit adenylate cyclase. Consequently, the intracellular concentration of cAMP is reduced upon D2R activation. Thus, it can be assumed that the level of reduction of intracellular cAMP is proportional to the number of activated D2R on the cell membrane. We found that the D2R-mediated changes of intracellular cAMP were increased in cells pretreated with Amph and this effect was prevented by the D2R antagonist haloperidol (Figure 3). Again, this observation suggests that prolonged treatments with Amph increase the amount of functional D2R at the cellular membrane. We cannot exclude, though, the possibility that prolonged Amph treatments had a general effect on proteins expression at the cellular membrane. However, this is unlikely since we measured no change in the cell surface staining of Na^+^/K^+^ ATPase after 15-h Amph treatment.

It is tempting to speculate that Amph mediates its effects at the D2R by acting on different kinases and/or other enzymes in the cytoplasm as previously shown in cells expressing DAT (Lute et al., 2008; Wheeler et al., 2015). However, since we do not have data indicating that Amph, without the dopamine transporter finds its way into the cytoplasm, we cannot sufficiently support this claim in the present study. Alternatively, it is possible that Amph permeates but does not cross the cellular membrane and this would be enough to alter D2R distribution in the lipid bilayer. Future and more specific experiments will elucidate these different possibilities.

The most intuitive conclusion drawn from the present data would be that Amph directly binds to the D2R. However, this seems unlikely because Simmler et al. (2013) have already shown that Amph exhibits low binding affinity (*Ki* > 30 μM) to the hD2R in membrane preparations from HEK293 cells transfected with hD2R. Another important implication of our results is that Amph can potentially alter the dopaminergic signaling without dopamine. This is an important consideration in situations where dopamine depletion occurs, for example, during repeated exposures to high concentrations of Amph (Wagner et al., 1980; Atianjoh et al., 2008).

In conclusion, we have provided evidence that in cells expressing hDAT, prolonged treatments with 1 or 50 μM Amph decreased the endogenous hD2R, whereas, in cells lacking hDAT, Amph increased hD2R at the cellular membrane. Because our results were collected from cells naturally expressing hD2R, and because the concentrations of Amph we used are relevant for human health, we believe our data might be of physiological relevance.

## Data Availability Statement

The original contributions presented in the study are included in the article/supplementary material, further inquiries can be directed to the corresponding author.

## Author Contributions

LC designed the study, analyzed the data, and wrote the manuscript. VN, SPM, FPM, and ZG designed and performed the experiments. AM quantified WB data. All authors contributed to the article and approved the submitted version.

## Conflict of Interest

The authors declare that the research was conducted in the absence of any commercial or financial relationships that could be construed as a potential conflict of interest.

## Publisher’s Note

All claims expressed in this article are solely those of the authors and do not necessarily represent those of their affiliated organizations, or those of the publisher, the editors and the reviewers. Any product that may be evaluated in this article, or claim that may be made by its manufacturer, is not guaranteed or endorsed by the publisher.
